# Comparing the Effectiveness of Digital and Conventional Rehabilitation Aftercare on Work Ability in Orthopedic Patients: A Longitudinal Study in Germany

**DOI:** 10.1007/s10926-025-10284-5

**Published:** 2025-03-27

**Authors:** Detlef Schmidt, Jakob Hedin, Anna Pelegrina, Susanne Weyland, Lena-Marie Rittmann, Darko Jekauc

**Affiliations:** 1 DRV Knappschaft-Bahn-See, Bochum, Germany; 2 Karlsruhe Institute of Technology, Karlsruhe, Germany

**Keywords:** Digital rehabilitation, Work ability, Orthopedic rehabilitation, Aftercare, Telerehabilitation

## Abstract

**Purpose:**

The primary aim of this study was to compare the effectiveness of digital rehabilitation aftercare (digIRENA) with conventional rehabilitation aftercare (IRENA) and a control group without organized aftercare in improving work ability among orthopedic patients.

**Methods:**

A total of 1056 orthopedic rehabilitation patients were recruited from multiple rehabilitation clinics in Germany and randomly assigned to three groups: digIRENA (*n* = 405), IRENA (*n* = 352), or a control group (*n* = 299). Work ability was assessed using the short version of the Work Ability Index at four time points: baseline, 13, 26, and 43 weeks post-baseline. Repeated-measures ANOVA was conducted to examine longitudinal trends in work ability, with additional analyses to assess the impact of age, gender, and employment status on outcomes.

**Results:**

Work ability improved significantly over time in all three groups (F = 37.6, *p* < 0.01, *η*^2^ = 0.045). In the unadjusted analysis, the interaction between time and group was significant (F = 2.2, *p* < 0.01, *η*^2^ = 0.006), indicating a steeper initial improvement in the digIRENA group compared to IRENA and control. However, when adjusting for age, gender, and employment status, this difference was no longer significant, suggesting that selection bias and baseline differences explain the unadjusted group effect. Across all groups, younger and unemployed participants showed greater improvements in work ability.

**Conclusion:**

In unadjusted comparisons, digital aftercare showed a steeper initial improvement in work ability. However, once key sociodemographic factors were controlled for, these group differences disappeared.

## Introduction

Orthopedic rehabilitation, also referred to as rehabilitation for musculoskeletal diseases (MSDs), plays a crucial role in the recovery and reintegration of individuals affected by musculoskeletal disorders, fractures, joint replacements, and other orthopedic conditions [1]. Globally, MSDs are leading contributors to work disability, affecting millions of individuals each year [2]. Orthopedic impairments often result in functional limitations that restrict an individual’s ability to return to work, thereby affecting both personal livelihoods and broader economic productivity [3]. Beyond economic implications, work is recognized as an important factor in promoting physical health, psychological well-being, and social inclusion [4]. Returning to work after rehabilitation has been shown to improve self-esteem, reduce symptoms of depression, and enhance overall quality of life [5]. Rehabilitation programs are thus essential, not only for improving physical functioning but also for restoring work ability, which is critical for maintaining labor force participation.

Orthopedic conditions, encompassing a wide range of MSDs, are particularly significant in Germany, contributing to 12% of long-term sick leave and early retirement [6]. MSD-related disabilities contributed to 888.9 million work disability days in 2022, resulting in an estimated €207 billion loss in gross value added and €118 billion in economic production losses [7]. As the number of individuals aged 67 and above in Germany is projected to rise by 22%, from 16 million in 2020 to 20 million by 2035 [8], preserving work ability through effective rehabilitation becomes an increasingly critical societal challenge. This age threshold is significant because 67 marks the standard retirement age in Germany, and ensuring work ability for those approaching or extending beyond this age is essential for maintaining workforce participation and addressing the challenges of an aging population.

In Germany, medical rehabilitation is an integral part of the healthcare system, with primary responsibility resting on the Deutsche Rentenversicherung (German Pension Insurance) for individuals of working age. This model emphasizes the principles of “rehabilitation before pension” to enable patients to reintegrate into the workforce and “rehabilitation before care” to prevent dependency on long-term care services [9]. Rehabilitation services typically encompass a three-week inpatient or outpatient program focusing on restoring physical and functional capacity, followed by tailored aftercare interventions to sustain and build upon the initial rehabilitation outcomes [10]. The majority of orthopedic rehabilitation programs target musculoskeletal conditions such as osteoarthritis, back pain, fractures, and joint replacements [11], which are among the leading causes of work disability in Germany [12].

In Germany, medical rehabilitation is highly structured and aims to achieve measurable improvements in functional capacity, quality of life, and work ability [13, 14]. Evidence from international systematic reviews highlights the effectiveness of multidisciplinary rehabilitation programs in improving physical functioning [15], mental health [16], and social reintegration [17] across various patient populations. These findings align with a meta-analytic results from German rehabilitation studies, which demonstrate substantial pre-post improvements in functional outcomes, particularly for musculoskeletal and psychosomatic conditions [14]. However, the meta-analysis emphasized the importance of tailoring rehabilitation interventions to patient-specific needs and the variability of outcomes across disease groups and patient demographics.

The restoration of work ability, defined as the balance between an individual’s resources and the demands of their work environment [18], is a central goal of rehabilitation interventions. Work ability declines with age [19], posing an increasing challenge in light of demographic changes. Reduced work ability is associated with higher risks of early retirement [20] and long-term sickness absence [21], making it a key determinant of both individual and societal well-being. Consequently, the German healthcare system, particularly through the German Pension Insurance, has implemented a range of rehabilitation interventions aimed at maintaining and improving work ability.

Although traditional rehabilitation programs, such as the intensive rehabilitation aftercare (IRENA) program, have demonstrated effectiveness in improving functional outcomes and reducing disability days [22], there is growing interest in the potential utility of digital rehabilitation platforms. Digital rehabilitation may offer a spatially accessible and flexible alternative to conventional approaches, addressing the challenges of motivation and adherence that often arise after the completion of in-person rehabilitation programs [23]. Studies have shown that rehabilitation has a positive effect on reducing early retirement and long-term sickness absence [24]. However, the impact of digital rehabilitation on work ability remains underexplored, and its effectiveness compared to conventional aftercare programs such as IRENA is not yet well established.

Given the significant socioeconomic implications of maintaining work ability and the potential for digital rehabilitation to enhance recovery, the current study aims to compare the effectiveness of digital rehabilitation aftercare with traditional IRENA and a control group on work ability in patients undergoing orthopedic rehabilitation.

## Methods

### Study Design

The study employed a quasi-experimental, longitudinal design to evaluate the impact of two rehabilitation aftercare approaches—digital and conventional—on work ability of orthopedic patients. Participants were allocated into three distinct groups: those receiving traditional aftercare, those enrolled in a digital aftercare program, and a control group without structured aftercare [25]. Data were collected at four intervals to monitor changes in work ability over time: at baseline (T0), midway through aftercare (T1), upon completion of the intervention (T2), and four months post-intervention (T3). This timeline was structured to allow for the assessment of both immediate and sustained effects of the rehabilitation interventions on work ability. The study was officially registered with the German Register of Clinical Studies under the identifier DRKS00022467. Ethical approval for the study was obtained from the Ethics Committee of the Karlsruhe Institute of Technology, and data protection compliance was ensured with the guidance of the Data Protection Officer at the Karlsruhe Institute of Technology. All study procedures adhered to the relevant ethical guidelines, and informed consent was obtained from all participants prior to their inclusion in the study.

### Sample

Power calculations indicated that a sample of 573 participants was required to assess the effectiveness of telerehabilitation aftercare, assuming a small effect size (Cohen’s f = 0.07) [26]. This calculation was performed with a significance level (α) of 0.05 and a statistical power of 0.80, suitable for repeated-measures ANOVA [25]. To account for an expected dropout rate of approximately 30%, the target recruitment was set at 1150 participants. However, due to a lower-than-anticipated dropout rate, 1056 orthopedic rehabilitation patients were enrolled at baseline.

A total of 1056 participants were recruited in 2020–2022 at several rehabilitation clinics, including those within the German Knappschafts-Kliniken network located in Badenweiler, Marquartstein, Bad Soden-Salmünster, Bad Homburg, and Warmbad. Participants were stratified into three groups: 405 in the digital rehabilitation group (digIRENA), 352 in the conventional rehabilitation group (IRENA), and 299 in the control group. Group allocation was partially randomized, taking into account participant preferences and availability of rehabilitation options. All participants were initially offered enrollment in the conventional IRENA program, which is the standard aftercare program in Germany. Participants who accepted this offer formed the non-randomized IRENA group. Those who declined participation in IRENA due to logistical or personal reasons were subsequently randomized in a 1:1 ratio to either the digIRENA group or the control group. However, a significant proportion of participants refused to participate in the study when they were allocated to the control group, resulting in smaller group sizes for the control group compared to the digIRENA group. This discrepancy highlights the challenge of maintaining balanced group sizes in a real-world setting, where patient preferences play a critical role. Despite these differences, randomization ensured comparability between the digIRENA and control groups in terms of baseline characteristics. Demographically, the majority of participants were male (67.3%), with an average age of 54.18 years. Retention rates across the study were robust, with 907 participants completing the second time point (T1) and 853 completing the final time point (T3).

## Interventions

### Traditional IRENA Aftercare

The traditional IRENA (Intensified Rehabilitation Aftercare) program is a structured multimodal aftercare intervention designed to support patients’ transition back into everyday life and work following orthopedic rehabilitation. It provides up to 24 sessions over a period of 12 months, with each session lasting between 90 and 120 min, depending on patient needs and rehabilitation goals. The program emphasizes maintaining and reinforcing the gains made during primary rehabilitation, focusing on enhancing physical, cognitive, and psychological well-being. Participants in the IRENA group were asked at follow-up time points (T1, T2, and T3) whether they had started the aftercare sessions, the number of weeks in which they participated in at least one session, and the average duration of their weekly participation. These self-reported data were used to estimate adherence to the program.

IRENA includes various therapeutic elements such as physical therapy, psychoeducation, and training sessions aimed at improving overall functional capacity, particularly in the musculoskeletal system [27]. The program is interdisciplinary, involving physiotherapists, psychologists, and social workers to deliver a comprehensive rehabilitation approach​. Sessions typically take place in a group setting, which allows participants to engage in supervised activities such as strength training, endurance exercises, and coordination activities. Additionally, psychoeducational components address stress management, coping strategies, and the integration of rehabilitation success into daily life​ [27].

### Digital IRENA (digIRENA)

The digital IRENA (digIRENA) program is a telerehabilitation aftercare intervention delivered through a digital platform, Caspar Health. The intervention is designed to provide flexible, remote access to rehabilitation services, allowing participants to continue their recovery independently, while still receiving structured guidance and support. The app offers tailored rehabilitation programs based on individual patient needs, similar to the traditional IRENA, but in a more accessible and user-driven format. The digIRENA platform delivers a combination of video-based exercises, educational content, and interactive features to facilitate the rehabilitation process. Patients can access personalized exercise programs, which include strength training, coordination exercises, and mobility activities, all of which are guided by video tutorials. These exercise modules are customized to each patient’s condition and rehabilitation goals and are updated periodically based on patient progress.

A key component of digIRENA is the remote supervision feature. Patients can submit progress reports, complete feedback forms, and communicate with their rehabilitation team via the app. Healthcare professionals, including physiotherapists and rehabilitation specialists, monitor the patient’s progress remotely and provide feedback or modifications to the exercise programs as needed. This interaction ensures that the rehabilitation process remains aligned with the patient’s recovery goals, even in a digital environment. In addition to the physical exercise components, psychoeducational content is delivered through the app to address mental health aspects such as stress management, motivation, and coping strategies. Patients receive regular notifications and reminders to keep them engaged and help maintain adherence to the rehabilitation plan. The app also incorporates tools for self-monitoring, enabling participants to track their progress over time and assess their work ability improvements. Adherence in the digIRENA group was tracked through self-reported measures, including the number of weeks participants engaged with the program and the average weekly time spent on exercises and activities within the platform. These measures were captured at T1, T2, and T3 to calculate overall adherence.

#### Control Group

In the control group, no structured aftercare intervention was offered. Participants were encouraged to engage in self-directed physical activity, but adherence to this recommendation was not formally tracked. Unlike those in the IRENA or digIRENA groups, control group members were encouraged to engage in self-directed physical activity but were not provided with formal support, guidance, or monitoring from rehabilitation professionals. This allowed for a comparison between structured rehabilitation aftercare and no formalized follow-up, helping to assess the effectiveness of both digital and conventional aftercare interventions on work ability.

## Measures

### Work Ability

The primary outcome of the study was work ability, assessed using the short version of the Work Ability Index (WAI) developed by Hasselhorn and Freude [28]. The WAI is a validated tool that measures work ability including seven aspects: (I) current work ability compared to lifetime best, (II) work ability in relation to job demands, (III) number of physician-diagnosed diseases, (IV) self-assessed impairment of work ability due to diseases, (V) sick leave days in the past 12 months, (VI) self-perceived ability to work over the next two years, and (VII) mental performance reserve. The WAI consists of 10 items with both categorical and continuous response formats, generating a total score that ranges from 7 to 49. Higher scores indicate better work ability. The WAI has demonstrated good reliability, with a Cronbach’s alpha of 0.78 [29], and its validity is supported by its predictive ability regarding early career exit and the duration of work disability [30, 31]. In addition to the WAI, demographic data such as age, gender, and employment status were collected through the questionnaire. The employment status variable was categorized into three groups: full-time, part-time, and unemployed. These demographic variables were used to control for potential confounding effects when analyzing the impact of the interventions on work ability.

### Adherence

Adherence to the rehabilitation aftercare programs was assessed through self-reported measures collected at three follow-up time points (T1, T2, T3). Participants in both the digIRENA and IRENA groups were asked whether they had started their respective aftercare programs. For those who had initiated participation, additional information was collected on the frequency of participation (number of weeks in which at least one session was attended) and the average weekly duration of participation (in minutes). These data allowed for the calculation of the total training time for each participant at each time point.

### Statistical Analyses

Prior to conducting statistical analyses, the dataset underwent preliminary data processing. Missing data were analyzed separately for each measurement point using Little’s MCAR test to determine if the data were missing completely at random [32]. Missing values at the item level were handled through an Expectation Maximization algorithm, which iteratively calculates maximum likelihood estimates. Post-imputation, imputed values were rounded to the nearest integer. If fewer than 50% of the required data for a specific questionnaire were available, missing data were not imputed, and the scale score was recorded as missing, thereby excluding the data from further analysis.

Descriptive statistics were computed for the variables gender, age, nationality, and employment status. Differences between the IRENA and digIRENA groups in terms of gender, nationality, and employment status were assessed using chi-squared goodness-of-fit tests, while one-way ANOVA was employed to analyze differences in age between the groups. Additionally, dropout analyses were conducted to determine whether the missingness was random or systematic using Little’s MCAR test [32]. Although the IRENA group was not randomized, it was included in the analysis to serve as a comparator group, reflecting real-world practices where patients often choose conventional rehabilitation. The inclusion of this group provides a context for evaluating the effectiveness of the digIRENA intervention and allows comparisons with the standard of care. To account for potential biases introduced by the non-randomized nature of the IRENA group, baseline characteristics were compared across groups, and differences were controlled for in the analyses where applicable. This approach aimed to reduce the influence of group imbalances on the observed intervention effects.

Adherence to the rehabilitation aftercare interventions was analyzed descriptively, based on self-reported data collected at T1, T2, and T3. Participants in both the digIRENA and IRENA groups reported whether they had started the aftercare intervention and, if so, provided information on the number of weeks they had participated in at least one session and their average weekly participation time. From these data, the total adherence time in minutes was calculated. Differences in adherence between the digIRENA and IRENA groups were assessed using chi-squared tests for the initiation of aftercare participation and independent samples t-tests for the total adherence time at each measurement point. Effect sizes (Cohen’s d and φ) were reported for group comparisons to provide a measure of the magnitude of these differences.

To evaluate changes in work ability over time, repeated-measures ANOVA was conducted across the four measurement points (T0, T1, T2, T3). This analysis facilitated the examination of longitudinal trends in work ability and differences between the intervention groups. Mauchly’s test was used to assess the assumption of sphericity, and if violations were detected, the Greenhouse–Geisser correction was applied.

A further repeated-measures ANCOVA was conducted to assess the interaction between time and group, age, gender, and employment status, following previous findings that suggest age-related differences in responses to digital and conventional rehabilitation approaches. For the analysis of age-related effects, participants were categorized into younger and older age groups using a median split. Mean trajectories of work ability across the four time points were presented graphically with 95% confidence intervals for each group. In cases where significant interactions were identified, additional graphical analyses were provided. The selection of repeated-measures ANOVA and ANCOVA as the primary analytical approaches was guided by the longitudinal nature of the data and the research objective of evaluating changes in work ability over time. These methods are well suited for analyzing within-subject changes and time-dependent interactions, while accounting for repeated measures on the same individuals. Repeated-measures ANOVA provides a robust framework for assessing group differences over time, while ANCOVA enables the inclusion of covariates to control for baseline differences and examine potential moderators such as age, gender, and employment status. All analyses were performed in SPSS version 28, using an alpha level of 0.05.

## Results

### Sample Description and Dropout Analyses

At baseline (T0), a total of 1056 participants were enrolled in the study, with 405 participants assigned to the digIRENA group, 352 to the IRENA group, and 299 to the control group (see Table 1). The overall sample was predominantly male, comprising 67.3% of the total population, with a similar proportion of males in the digIRENA (64.2%) and IRENA (65.6%) groups. However, the proportion of male participants was significantly higher in the control group (73.6%) compared to the other two groups, as indicated by a chi-square test (*χ*^2^ = 7.5, df = 2, *p* = 0.024). The mean age of participants significantly differed between the groups (F = 10.5, df1 = 2, df2 = 1052, *p* < 0.01, *η*^2^ = 0.020). Participants in the control group were on average slightly older (M = 55.74, SD = 6.30) compared to the IRENA group (M = 54.21, SD = 7.90) and the digIRENA group (M = 53.00, SD = 8.75). Bonferroni post hoc analysis showed that the mean age in the control group was significantly higher than in both the IRENA and digIRENA groups. Most participants were of German nationality (97.6%). There were no significant differences in nationality distribution between the groups (*χ*^2^ = 3.0, df = 2, *p* = 0.224). Regarding employment status, 77.7% of participants were employed full-time, 12.0% part-time, and 9.6% were unemployed. No significant differences were observed between the groups in terms of employment status (*χ*^2^ = 4.2, df = 4, *p* = 0.374).

**Table 1:** Sample characteristics at T0

	digIRENA	IRENA	Control	Overall
	405 (100%)	352 (100%)	299 (100%)	1056 (100%)
Gender				
Male	260 (64.2%)	231 (65.6%)	220 (73.6%)	711 (67.3%)
Female	145 (35.8%)	120 (34.1%)	79 (26.4%)	344 (32.6%)
Divers	0	1 (0.3%)	0	1 (0.1%)
Missing	0	0	0	0
Age				
Mean	53.00	54.21	55.74	54.18
Standard deviation	8.75	7.90	6.30	7.91
n	405	351	299	1055
Missing	0	1	0	1
Nationality				
German	398 (98.3%)	339 (96.3%)	294 (98.3%)	1031 (97.6%)
Non-German	7 (1.7%)	11 (3.1%)	4 (1.3%)	22 (2.1%)
Missing	0	2 (0.6%)	1 (0.3%)	3 (0.3%)
Employment status				
Full-Time (> 34 h)	318 (78.5%)	265 (75.3%)	238 (79.6%)	821 (77.7%)
Part-Time (< 34 h)	53 (13.1%)	45 (12.8%)	29 (9.7%)	127 (12.0%)
Education	2 (0.5%)	2 (0.6%)	0	4 (0.4%)
Unemployed	32 (7.9%)	37 (10.5%)	32 (10.7%)	101 (9.6%)
Missing	0	3 (0.9%)	0	3 (0.3%)

Dropout rates were assessed across the four measurement time points (T0, T1, T2, and T3). At baseline, 33 participants (3.13% of the total sample) had missing data for the WAI, affecting a total of 73 data points (0.69%) across 10 of the 24 items. Little’s MCAR test indicated that the missing data at T0 were completely random (*χ*^2^ = 160.4; df = 144; *p* = 0.165). At T1, 22 participants (2.43% of the remaining sample of 907) had missing data for the WAI, affecting 41 values (0.45%) across the same 10 items. The MCAR test again suggested that the missing data were random (*χ*^2^ = 101.6; df = 102; *p* = 0.491). At T2, 15 participants (1.73% of the 866 remaining participants) had missing WAI data, impacting 52 values (0.60%). Little’s test indicated no systematic pattern in the missing data (*χ*^2^ = 65.4; df = 65; *p* = 0.464). By T3, 19 participants (2.23% of the 853 remaining participants) had missing data, with 30 values (0.35%) missing across 10 WAI items. As with the previous time points, the missing data were deemed to be completely random (*χ*^2^ = 93.0; df = 93; *p* = 0.481). These analyses suggest that no significant patterns of systematic missingness were observed.

### Adherence

At T1, 98.9% of participants in the digIRENA group reported initiating the program compared to 77.5% in the IRENA group, with a significant difference in proportions (*χ*^2^ = 78.3; df = 1; *p* < 0.01; φ = 0.35). At T2, participation rates remained higher in the digIRENA group (98.9%) than in the IRENA group (86.6%) (*χ*^2^ = 38.6; df = 1; *p* < 0.01; φ = 0.25). Similarly, at T3, 99.5% of the digIRENA participants had engaged with the program, compared to 88% in the IRENA group, demonstrating a consistent advantage for digIRENA across all time points (*χ*^2^ = 38.1; df = 1; *p* < 0.01; φ = 0.25).

The total reported training time, calculated in minutes, was significantly higher in the digIRENA group compared to the IRENA group at all time points. At T1, the average training time in the digIRENA group was 982.8 min (SD = 562.5) versus 789.1 min (SD = 676.5) in the IRENA group (t = 3.8; df = 522.2; *p* < 0.01; d = 0.32). At T2, participants in the digIRENA group reported an average of 1551.8 min (SD = 636.4) compared to 1314.7 min (SD = 798.5) in the IRENA group (t = 3.8; df = 447.5; *p* < 0.01; d = 0.33). By T3, the average training time in the digIRENA group further increased to 1836.2 min (SD = 545.8), significantly exceeding the IRENA group’s 1491.9 min (SD = 825.8) (t = 5.1; df = 366.8; *p* < 0.01; d = 0.50).

### Work Ability

The descriptive statistics for the WAI across the four measurement points (T0, T1, T2, T3) are presented in Table 2. At baseline (T0), mean WAI scores were similar among the three groups with no significant differences between the three groups (F = 2.2; df_1_ = 2; df_2_ = 1048; *p* = 0.11).

**Table 2:** Descriptive statistics for WAI

Time	digIRENA			IRENA			Control		
	n	Mean	SD	n	Mean	SD	n	Mean	SD
T0	319	27.94	7.63	249	27.19	7.79	230	28.39	7.51
T1	319	29.95	7.86	249	28.63	9.00	230	29.51	8.30
T2	319	30.58	8.18	249	28.87	9.10	230	29.50	8.60
T3	319	30.63	8.57	249	28.91	9.36	230	29.60	9.04

*n* Number of participants, *SD* standard deviation

**Fig. 1:**
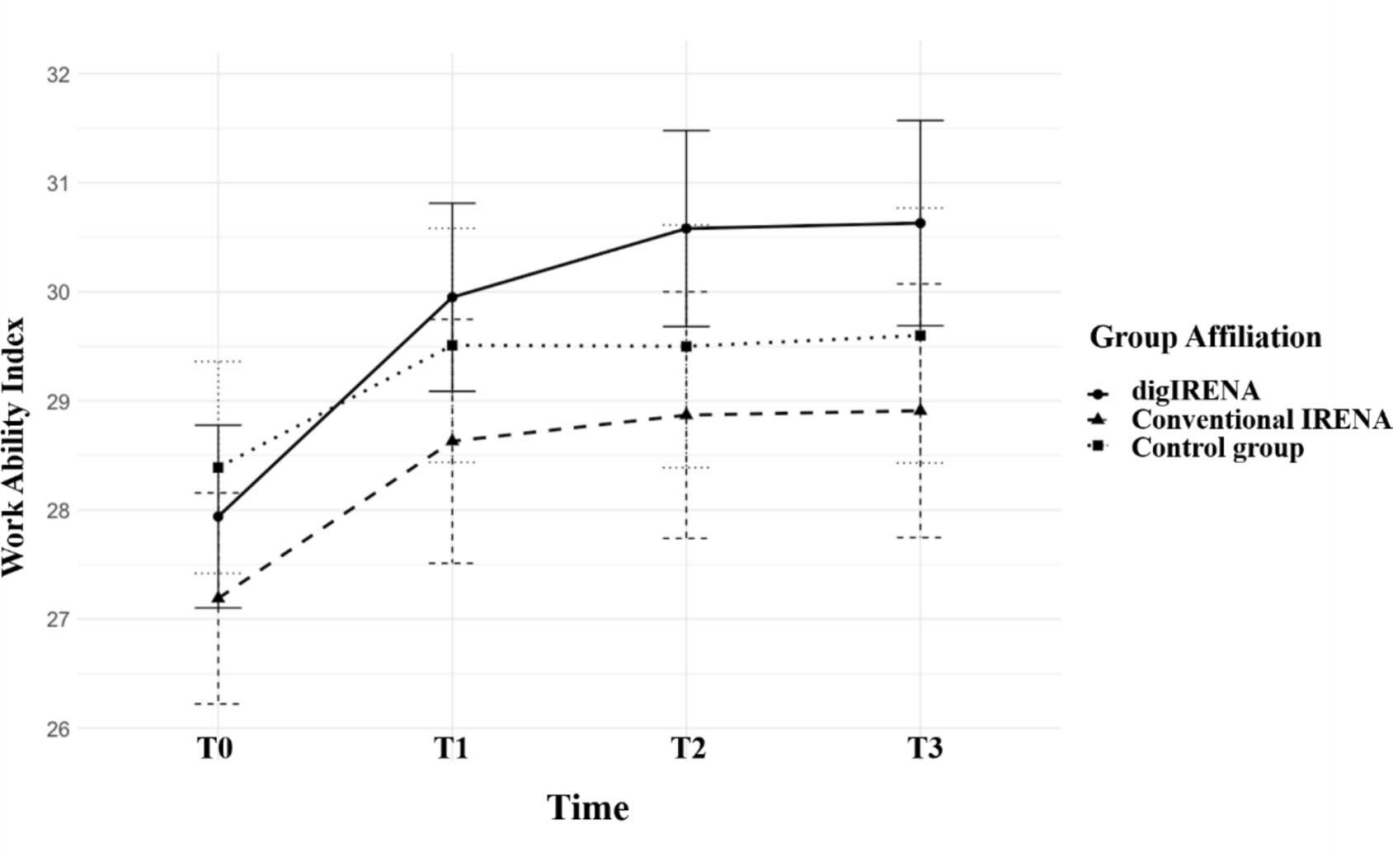
Development of WAI for digIRENA, IRENA, and control groups from T0 to T3

The results of the analysis of variance with repeated measurement are presented in Table 3 and indicate that both time (F = 37.6, df_1_ = 2.7, df_2_ = 2113.4, *p* < 0.01, *η*^2^ = 0.045) and the interaction between time and group (F = 2.2, df_1_ = 5.3, df_2_ = 2113.4, *p* < 0.01, *η*^2^ = 0.006) had significant effects on work ability. The significant effect of time suggests that all three groups showed significant improvements in work ability over time. Further analyses revealed both a linear (F = 58.4, df_1_ = 1, df_2_ = 18,642.6, *p* < 0.01, *η*^2^ = 0.068) and a quadratic (F = 28.7, df_1_ = 1, df_2_ = 11,552.4, *p* < 0.01, *η*^2^ = 0.035) trend in the development of work ability over time. The increase in work ability from T0 to T1 was relatively steep for all groups, after which the rate of improvement plateaued between T1 and T2 (see Fig. 1). Between T2 and T3, the mean work ability scores remained stable across all groups. The unadjusted repeated-measures ANOVA revealed a significant Time × Group interaction (F = 3.9, df_1_ = 2, df_2_ = 18,642.6, *p* = 0.02, *η*^2^ = 0.010), with the digIRENA group displaying a steeper improvement in work ability initially (T0 to T2) (see Fig. 1). However, in an adjusted model including age, gender, and employment status, this Time × Group interaction was no longer significant, indicating that the raw group differences are more likely driven by baseline confounders rather than a distinct effect of the digital intervention.

**Table 3:** ANOVA with repeated measurement with time and group as independent variables

Variable	SS	df	MSS	F	p	Partial *η*^2^
Time	1825.1	2.7	686.5	37.6	< 0.001	0.045
Time*Group	214.0	5.3	40.3	2.2	0.047	0.006
Error	38,564.2	2113.4	18.2			

*SS* sum of squares, *df* degrees of freedom, *MSS* mean sum of squares

### Analyses of Variance with Age, Gender, and Employment Status

In a subsequent analysis, gender and employment status were included as factors, while age was treated as a covariate in the repeated-measures ANOVA. The results are presented in Table 4. The effect of time remained significant (F = 26.7, df_1_ = 2.7, df_2_ = 2092.8, *p* < 0.001, *η*^2^ = 0.033), while the interaction between time and group (F = 0.7, df_1_ = 5.4, df_2_ = 2092.8, *p* = 0.608, *η*^2^ = 0.002) was no longer significant. However, significant interactions were observed between time and age (F = 16.8, df_1_ = 2.7, df_2_ = 2092.8, *p* < 0.001, *η*^2^ = 0.021) and between time and employment status (F = 4.3, df_1_ = 5.4, df_2_ = 2092.8, *p* < 0.001, *η*^2^ = 0.011), indicating that these factors significantly influenced the development of work ability over the four measurement points.

**Table 4:** ANCOVA with repeated measurement including time, age, gender, group, and employment status as independent variables

Variable	SS	df	MSQ	F	p	Partial *η*^2^
Time	1248.9	2.7	461.3	26.7	< .001	0.033
Time*age	787.0	2.7	290.7	16.8	< .001	0.021
Time*group	68.8	5.4	12.7	0.7	0.608	0.002
Time*gender	1.7	2.7	0.6	0.0	0.986	0
Time*employment	405.6	5.4	74.9	4.3	< .001	0.011
Time*group*gender	52.9	5.4	9.8	0.6	0.741	0.001
Time*group*employment	256.2	10.8	23.7	1.4	0.183	0.007
Time*gender*employment	105.4	5.4	19.5	1.1	0.345	0.003
Time*group*gender*employment	86.3	10.8	8.0	0.5	0.926	0.002
Error	36211.1	2092.8	17.3			

*SS* Sum of squares; *df* degrees of freedom, *MSS* mean sum of squares

**Fig. 2:**
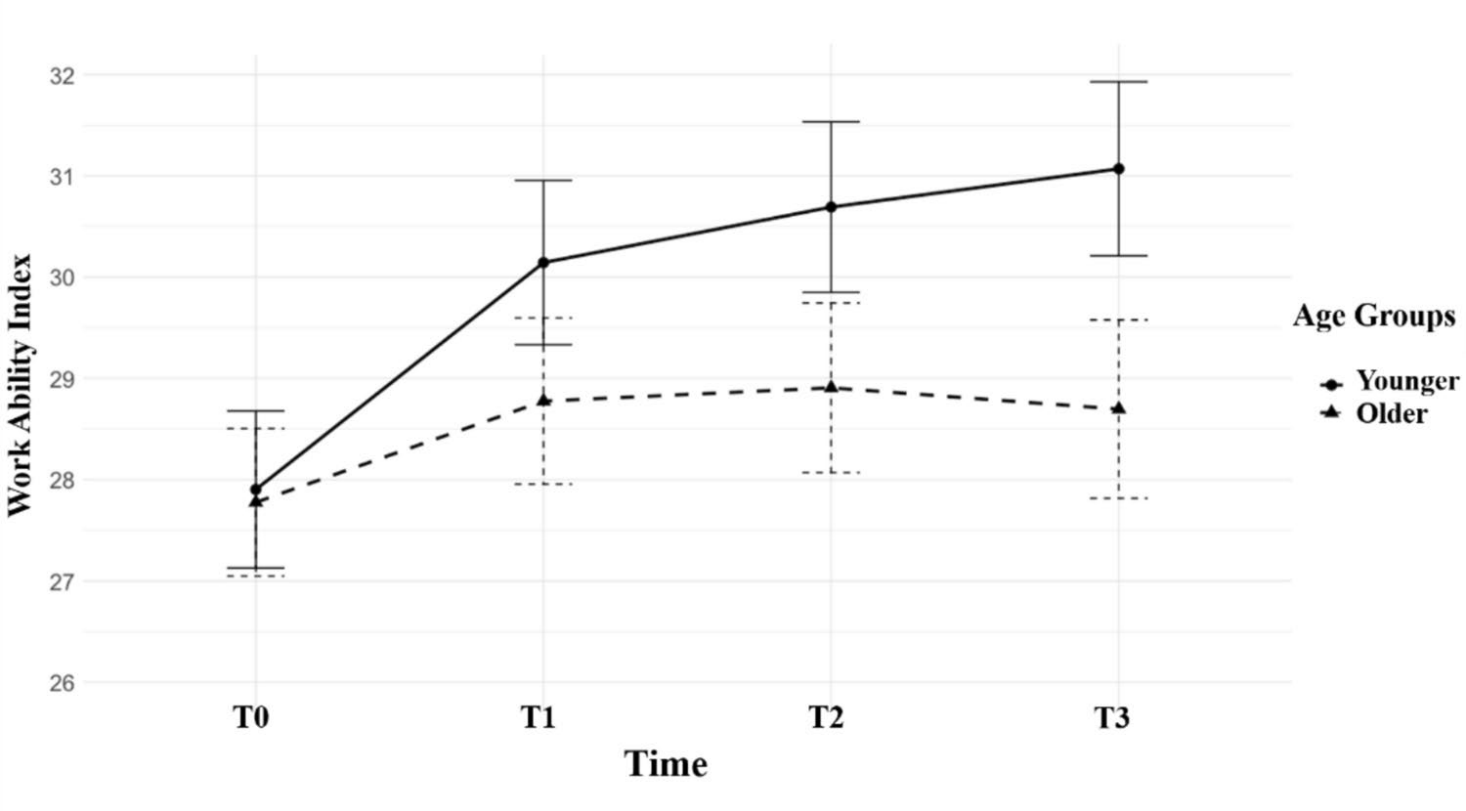
Development of WAI for younger (≤ 55 years) and older (> 55 years) participants from T0 to T3

**Fig. 3:**
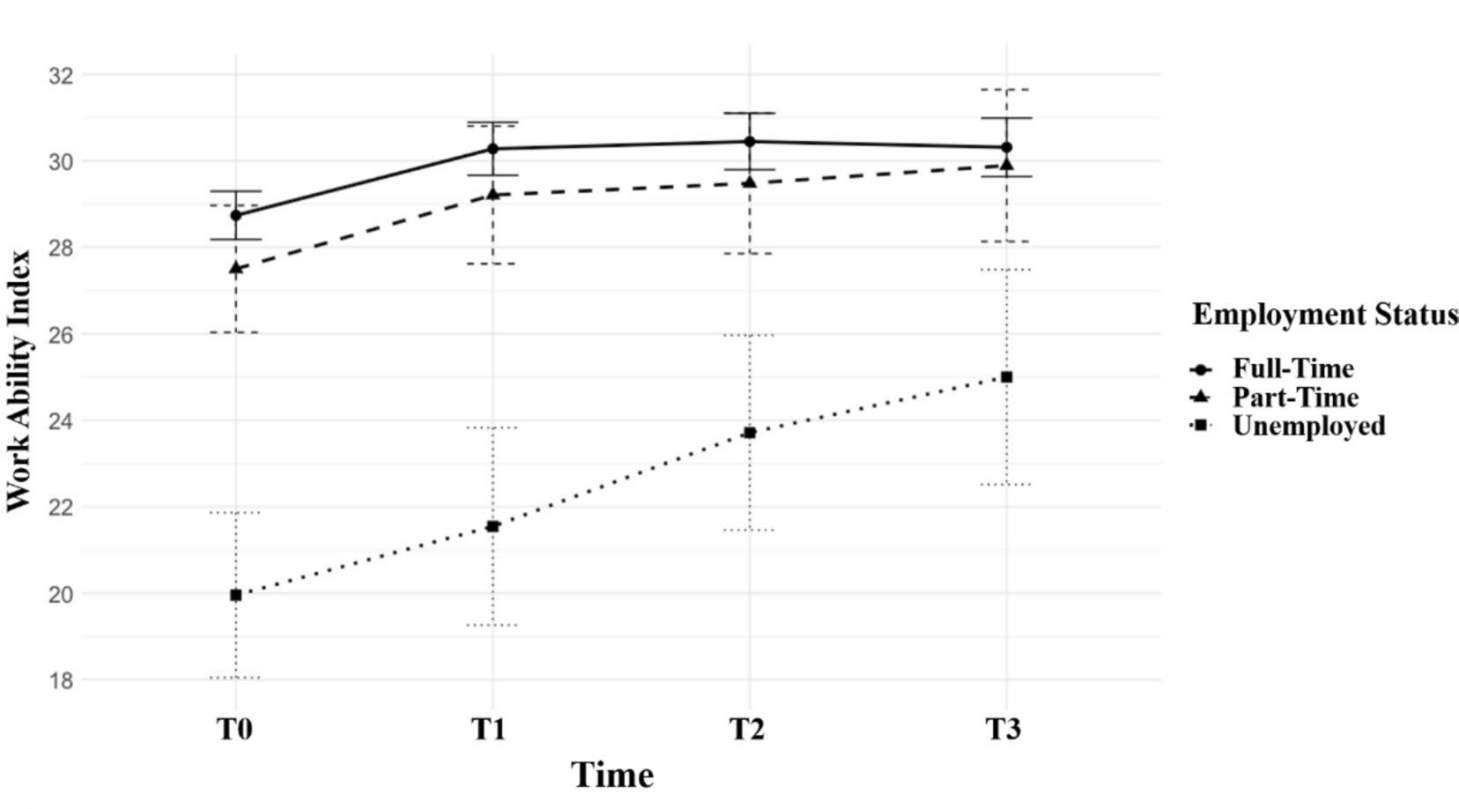
Development WAI based on employment status from T0 to T3

In a further analysis, age was dichotomized using a median split, and the repeated-measures ANOVA was recalculated. The results indicated that younger participants (aged 55 years and younger) benefited more significantly from the rehabilitation intervention compared to older participants (see Fig. 2). Additionally, the interaction between time and employment status revealed that non-working individuals had a significantly lower baseline level of work ability compared to those employed full-time or part-time (see Fig. 3). However, the unemployed group exhibited a steeper increase in work ability over the four measurement points, compared to the other two employment groups.

## Discussion

The primary objective of this study was to compare the effectiveness of digital rehabilitation aftercare with traditional IRENA and a control group in improving work ability among orthopedic patients. All three groups demonstrated improvements in work ability over time in the unadjusted analysis. While digIRENA initially appeared to show a steeper and faster improvement, the Time × Group interaction was not significant once we controlled for age, gender, and employment status. This finding suggests that the apparent advantage in the unadjusted results may reflect selection biases or baseline imbalances, rather than a true superiority of the digital intervention. Additionally, age and employment status significantly influenced work ability outcomes, with younger participants and non-working individuals benefiting the most from the rehabilitation interventions.

These findings should also be interpreted in light of the potential influence of baseline group differences, as the semi-randomized allocation process may not have fully mitigated pre-existing disparities between groups. Although key demographic variables such as age, gender, and employment status were included as covariates in the analyses, other unmeasured factors—such as socioeconomic background or prior rehabilitation history—may have contributed to the observed outcomes. The absence of comprehensive baseline data limits the ability to definitively attribute the intervention effects solely to the rehabilitation programs. This limitation underscores the need for future studies to adopt more rigorous designs, including fully randomized controlled trials or statistical techniques like propensity score matching, to better address potential confounding effects and enhance causal inferences regarding the effectiveness of digital rehabilitation interventions.

The results of this study demonstrate a significant time effect, accounting for 4.5% of the within-person variance in work ability. Both a linear and quadratic trend were observed over time. The linear trend indicates that all three groups experienced significant improvements in work ability during the 12-week period following discharge from orthopedic rehabilitation. This finding aligns with the previous studies, which have consistently shown that structured rehabilitation interventions lead to improvements in work ability and overall functional capacity in orthopedic patients [33, 34]. These increases in work ability suggest that orthopedic rehabilitation, regardless of the mode of delivery, is generally effective in helping patients recover their capacity to work.

The quadratic trend observed in the data indicates that the improvement in work ability began to stabilize 12 weeks post-rehabilitation, with this stabilization continuing through the four-month follow-up period. This plateau suggests that the positive effects of rehabilitation are maintained over time, reflecting the long-term benefits of aftercare interventions. These findings are consistent with the previous research, which has shown that improvements in work ability tend to plateau after the initial phase of recovery, but can be sustained over several months with proper follow-up [35]. The sustainability of these effects is critical for preventing long-term work disability and early retirement, as supported by studies highlighting the long-term impact of rehabilitation on reducing sickness absence and improving workforce participation [34, 36].

In unadjusted analyses, the digIRENA group appeared to show both a steeper initial increase in work ability and continued gains during the second half of the follow-up period, while the IRENA and control groups stabilized. However, once we adjusted for sociodemographic factors (i.e., age, gender, and employment status), the apparent group effect was no longer evident. This suggests that the unadjusted differences might be more likely attributable to baseline imbalances rather than a true superiority of the digital intervention. Accordingly, these findings must be interpreted with caution, emphasizing the importance of rigorous randomization or advanced statistical controls to accurately determine whether digital aftercare provides long-term benefits over other approaches.

This study highlights the critical role of adherence in maximizing the benefits of rehabilitation interventions. The self-reported data on adherence revealed that participants in the digIRENA group were more likely to initiate and sustain their engagement with aftercare programs compared to the traditional IRENA group. This difference in adherence may explain the superior outcomes observed in the digIRENA group, as consistent participation is essential for achieving optimal rehabilitation outcomes. The flexibility and accessibility of digital platforms likely contributed to higher adherence rates, allowing participants to integrate rehabilitation exercises into their daily routines more effectively [37]. However, future research should explore objective measures of adherence, such as platform usage logs, to validate self-reported data and provide deeper insights into the mechanisms driving these differences.

These findings are consistent with the previous studies that have reported benefits of digital rehabilitation platforms in maintaining patient engagement and adherence to rehabilitation protocols, particularly when compared to traditional rehabilitation [38]. The continued improvement observed in the digIRENA group may be attributed to the flexibility and accessibility of digital interventions, which allow patients to integrate rehabilitation exercises into their daily lives more easily. Similar results were found in a study by Martinez-Rico et al. [39], where telerehabilitation intervention led to sustained functional gains over time, surpassing traditional rehabilitation program. This suggests that digital rehabilitation platforms like digIRENA may play a key role in enhancing work ability, particularly for patients who face logistical barriers to accessing conventional rehabilitation services [40].

When age, gender, and employment status were included in the ANOVA, the interaction between time and group was no longer significant, suggesting that sociodemographic variables may have moderated this effect. Notably, the interaction between time and age emerged as a significant factor, indicating that younger participants benefited more from the rehabilitation interventions than their older counterparts. In younger participants, the increase in work ability was continuous and sustained, even after the formal aftercare period. In contrast, for older participants, improvements were only evident during the first 12 weeks of post-rehabilitation, with little or no further gains afterward.

This age-related difference in response to rehabilitation may be due to several factors. Biological aging processes, such as slower recovery rates, reduced physical resilience, and the presence of comorbidities, could explain the diminished gains observed in older individuals. Research has shown that as people age, physical recovery from musculoskeletal injuries or surgeries tends to be slower [41], which could limit the overall effectiveness of rehabilitation programs. Another possible explanation is related to digital literacy. Younger participants may have been more comfortable using the digital platform provided by the digIRENA intervention, which could have enhanced their engagement and adherence to the rehabilitation program. Studies have shown that digital literacy can influence the effectiveness of online health interventions [42, 43], with younger individuals generally being more adept at navigating and utilizing digital tools [44]. This may explain why the younger participants in our study showed more sustained improvements, particularly in the digital aftercare group. Similar findings were reported by Schmidt et al. [45], who noted that younger individuals tend to benefit more from digital prevention interventions.

The interaction between time and employment status had a significant effect on the development of work ability. Participants who were not employed at baseline had significantly lower initial levels of work ability compared to those who were employed either full-time or part-time. However, the non-employed group showed the steepest increase in work ability over time. This finding aligns with the previous research suggesting that individuals with lower initial functional capacity often experience the greatest relative gains from structured rehabilitation programs [46]. This suggests that individuals not currently in the workforce, possibly due to more severe health impairments, benefited the most from rehabilitation and aftercare interventions. One potential explanation for this finding is that non-employed individuals, likely facing greater functional limitations at baseline, had more room for improvement. The low initial levels of work ability could be attributed to more severe health problems, longer periods of inactivity, or other challenges associated with their conditions. Previous research has shown that individuals with more severe health issues often exhibit more pronounced physical and functional deficits but also tend to achieve greater relative gains from rehabilitation interventions [47]. The steep improvement in work ability in this group suggests that rehabilitation programs are particularly effective for those starting from a lower baseline, offering substantial potential to increase their self-acceptance, acceptance by others, physical capacity, psychological resources, and capacity to balance engagement [48].

Additionally, employment status may influence rehabilitation outcomes by shaping the time and resources participants can allocate to their recovery. Employed individuals may face competing priorities and time constraints, which could limit their adherence to aftercare interventions. Conversely, non-employed participants may have more flexibility to engage fully with rehabilitation programs, which could explain their steeper improvement trajectory [39]. However, research specifically addressing the role of employment status in rehabilitation outcomes is limited, and further studies are needed to explore how these factors interact to influence long-term work ability. Furthermore, the current findings highlight the need for more research on the relationship between employment status and rehabilitation outcomes, particularly in the context of digital aftercare interventions. While previous studies have examined general predictors of rehabilitation success [49, 50], the specific mechanisms through which employment status affects work ability remain underexplored. By demonstrating that non-employed individuals can achieve significant improvements in work ability, this study contributes novel insights to the field and underscores the importance of tailoring interventions to the needs of different employment groups.

## Implications for Practice

The findings of this study have several important implications for clinical practice and rehabilitation programs. First, although unadjusted analyses indicated higher initial gains for digital aftercare, this difference did not remain once demographic variables were controlled for. This underscores that rigorous randomization or adjusted designs are essential for reliably assessing the effectiveness of digital interventions. Digital platforms may nonetheless offer important logistical advantages and the potential for improved adherence, but further studies are needed to clarify whether these platforms confer a superior effect on work ability, independent of selection bias. The study also underscores the importance of tailoring rehabilitation programs to specific patient populations, particularly in terms of age and employment status. Younger patients showed more consistent improvement in work ability, suggesting that digital interventions may be particularly well-suited for this demographic, likely due to their higher digital literacy and comfort with technology. Conversely, older patients may require additional support, such as hybrid models combining in-person and digital care, to achieve similar outcomes. Rehabilitation programs should consider offering more personalized interventions for older adults to account for their slower recovery rates and potential challenges in engaging with digital platforms.

The findings also demonstrate that rehabilitation and aftercare interventions can have a substantial impact on improving work ability for unemployed individuals. These individuals, who may begin rehabilitation with lower functional levels, benefit significantly from structured rehabilitation programs. The significant improvements observed in non-employed individuals suggest that rehabilitation programs could serve as a platform for enhancing not only physical recovery but also employability. Future interventions might benefit from incorporating job readiness training or vocational support to complement physical rehabilitation, thereby addressing the multifaceted challenges faced by non-employed individuals. Finally, the study reinforces the importance of long-term follow-up care. The sustained improvements in work ability seen in the digital aftercare group indicate that ongoing support beyond the initial rehabilitation period is crucial for maintaining functional gains. Healthcare providers should consider implementing long-term digital or hybrid follow-up care models to support patients’ work ability and prevent relapses or declines in functional capacity.

## Limitations

Several limitations of this study should be acknowledged. First, the study relied on self-reported measures of work ability using the WAI, which may introduce reporting bias. Participants’ subjective perceptions of their work ability might not fully capture objective improvements in physical or functional capacity. Future studies would benefit from incorporating objective measures, such as functional tests or workplace performance evaluations, to provide a more comprehensive assessment of rehabilitation outcomes. Second, while the study sample was large and diverse, the majority of participants were male (67.3%), limiting the generalizability of the findings to other populations, particularly women. Comprehensive national statistics detailing the gender distribution within orthopedic rehabilitation in Germany are not available, making it unclear whether this gender proportion is representative of the broader orthopedic rehabilitation population. However, it is possible that the recruitment strategy or the nature of orthopedic rehabilitation referrals may have contributed to the overrepresentation of male participants.

Third, the allocation process introduced potential limitations. The IRENA group was non-randomized, as all participants were initially offered this standard aftercare program, and those who accepted were assigned to this group. This lack of randomization may limit direct comparability between the IRENA group and the other groups, which were assigned through a randomization process. Additionally, a significant proportion of participants declined to participate in the study when allocated to the control group, leading to a smaller sample size for this group. This high dropout rate in the control group could have introduced bias and reduced the statistical power to detect differences between the groups. Future studies should consider strategies to enhance participant retention, particularly in control groups, to ensure balanced and robust comparisons.

Fourth, adherence to the aftercare interventions was assessed through self-reported data, which may be subject to recall bias or social desirability bias. Objective measures of adherence, such as digital platform usage logs or attendance records for in-person sessions, were not available. Additionally, adherence to self-directed physical activity in the control group was not formally monitored, which limits the ability to account for potential contamination effects.

Fifth, another limitation of this study is the potential influence of observed and unobserved group differences. While demographic variables such as age, gender, and employment status were included as covariates in the analysis to mitigate these effects, the naturalistic design of the study means that additional factors—such as baseline health status, socioeconomic background, or prior rehabilitation history—may have contributed to the observed outcomes. The lack of comprehensive baseline data limits the ability to fully disentangle the effects of the interventions from pre-existing differences between groups. Future research should aim to incorporate a wider range of baseline variables and consider advanced statistical methods, such as propensity score matching or sensitivity analyses, to further address these concerns.

Lastly, the study was conducted within the German healthcare system, which may limit the external validity of the findings to other healthcare settings or countries with different rehabilitation practices or healthcare structures. Moreover, the follow-up period of four months may not have been sufficient to capture the long-term sustainability of the improvements in work ability. Future studies should consider extending the follow-up period to assess the long-term effects of rehabilitation interventions on work ability.

## Future Research Directions

Future research should focus on exploring the long-term sustainability of improvements in work ability following rehabilitation, particularly extending the follow-up period beyond four months to assess whether gains are maintained over several years. Additionally, studies should investigate the role of adherence and engagement with digital platforms like digIRENA to better understand how these factors influence the effectiveness of digital rehabilitation interventions. Future research should emphasize the role of health behavior maintenance in the post-rehabilitation phase. While structured aftercare interventions like digIRENA have proven effective in the short term, the long-term goal must be to encourage participants to engage in independent, self-initiated exercise and other health-promoting behaviors after the completion of formal rehabilitation programs.

Moreover, investigating the applicability of these findings across different healthcare systems and cultural contexts would provide valuable insights into the generalizability of digital rehabilitation interventions in diverse populations. Finally, future studies should explore hybrid models of rehabilitation that combine digital and in-person components, particularly for older adults or those less comfortable with technology, to optimize patient outcomes across varying demographic groups.

## Conclusion

This study found that both digital and traditional rehabilitation aftercare programs significantly improve work ability among orthopedic patients in unadjusted analyses, with the digital intervention (digIRENA) displaying the greatest observed gains. However, these results must be interpreted with caution due to the semi-randomized allocation process, baseline group differences, and the lack of comprehensive initial data. Once key demographic variables (e.g., age, gender, and employment status) were included, no definitive net benefit of digital rehabilitation aftercare over traditional aftercare or no aftercare could be established. This finding suggests that the observed group differences in unadjusted models likely result from selection bias rather than a true superiority of the digital intervention.

Nonetheless, this study underscores the potential of digital platforms to provide flexible and accessible rehabilitation solutions, particularly for patients who face logistical challenges in accessing traditional care. These platforms may be especially beneficial for younger individuals and those not currently employed, as these groups demonstrated the most substantial improvements. However, more rigorous research is needed to validate these results and to disentangle the effects of the interventions from confounding factors. Future studies should employ fully randomized controlled designs or advanced statistical methods, such as propensity score matching, to mitigate potential biases and strengthen causal inferences. Long-term evaluations that include objective measures of work ability, adherence, and health behaviors will also be critical to understanding the sustained impact of digital rehabilitation programs.

## Data Availability

The datasets used and/or analyzed during the current study are available from the corresponding author on reasonable request.
